# Near infrared spectroscopic data for rapid and simultaneous prediction of quality attributes in intact mango fruits

**DOI:** 10.1016/j.dib.2019.104789

**Published:** 2019-11-11

**Authors:** Agus Arip Munawar, Kusumiyati, Devi Wahyuni

**Affiliations:** a Department of Agricultural Engineering, Syiah Kuala University, Banda Aceh Indonesia; b Department of Agronomy, Padjadjaran University, Bandung Indonesia

**Keywords:** NIRS, Spectroscopy, Spectral data, Mango, Prediction

## Abstract

Presented dataset contains spectral data on near infrared region for a total of 186 intact mango fruit samples from 4 different cultivars (*cv. Kweni, Cengkir, Palmer* and *Kent*). Near infrared spectral data were collected and recorded as absorbance (Log(1/R)) data in wavelength range of 1000–2500 nm. Those spectral data are potential to be re-used and analysed for the prediction of mango quality attributes in form of vitamin C, soluble solids content (SSC) and total acidity (TA). Spectra data can be corrected and enhanced using several algorithms such as multiplicative scatter correction (MSC) and de-trending (DT). Prediction models can be established using common regression approach like partial least square regression (PLSR).

**Table:** Specifications Table

Subject	Agricultural and Biological Sciences
Specific subject area	Spectroscopy, non-destructive technique in agriculture.
Type of data	Table Graph Spectroscopic data
How data were acquired	Spectral datasets of all intact mango samples were acquired using a benchtop Fourier transform infrared spectroscopy (*Thermo Nicolet Antaris II TM*). Spectra range used were 1000–2500 nm with co-added of 64 scans and recorded as Log(1/R) absorbance spectra data.
Data format	Raw Analysed Enhanced Presented as *.xls* and *.unsb* file formats
Parameters for data collection	Data were collected for all 186 mango fruit samples with varied different maturity stages from un-ripen to senescence.
Description of data collection	Near infrared spectra data were collected for a total of 186 intact mango samples in wavelength range from 1000 to 2500 nm. On the other hand, actual vitamin C, SSC and TA of mango samples were measured using standard laboratory methods as follows: titration method used to measure Vitamin C and TA, expressed in mg.100g^−1^ fresh mass (FM); whilst refraction index was employed to acquire SSC data of mango samples and presented as ^o^Brix.
Data source location	Data were collected in Georg-August University of *Goettingen*, Germany.
Data accessibility	Dataset are available on this article and can be found in Mendeley data: https://doi.org/10.17632/b9d6s7hr33.1 or https://data.mendeley.com/datasets/b9d6s7hr33/1

**Table:** 

**Value of the Data** • Dataset obtained from Near infrared spectroscopy (NIRS) provide fast, non-destructive, simultaneous and pollution free to determine quality attributes of agricultural products like mango fruits. • These data can be used to develop prediction models used to predict vitamin C, soluble solids content (SSC) and total acidity (TA) of intact mangos without complicated sample procedures and preparations. • Data can be benefited for those who concentrated on rapid and non-destructive application for agriculture products. They can be from academics, agro-industries and practitioners. • Near infrared spectroscopy can be employed in foods and agricultural products industries especially for quality evaluations: sorting, grading and authenticating. • Prediction performances may vary, depends on spectra enhancement and regression approaches to be used.

## 1 Data

**Fig. 1:**
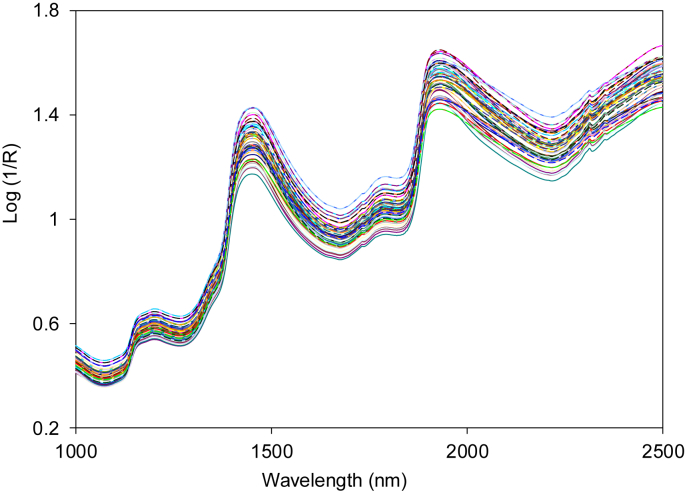
Near infrared spectrum of intact mangos before spectra enhancement (raw).

**Fig. 2:**
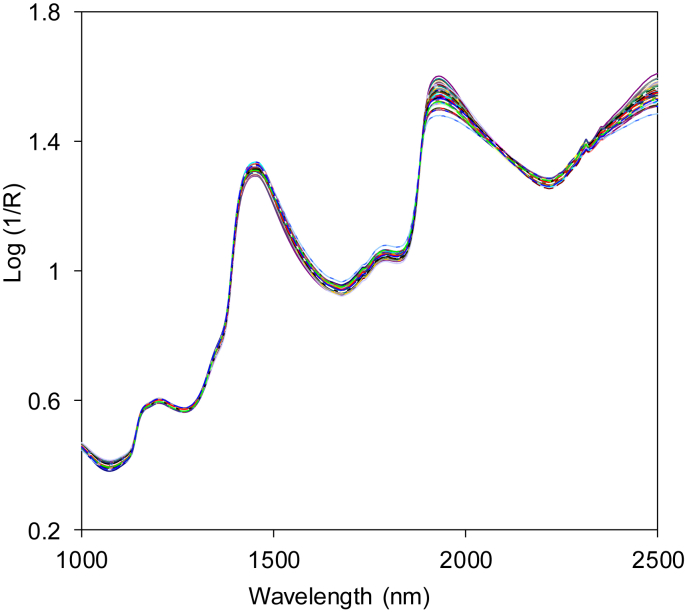
Near infrared spectrum after enhancement using multiplicative scatter correction (MSC).

**Fig. 3:**
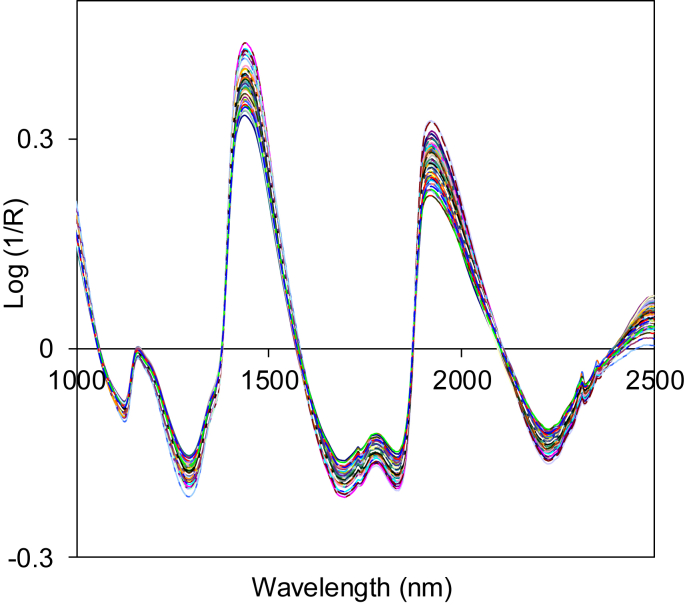
Near infrared spectrum after enhancement using de-trending approach (DT).

Near infrared spectral dataset acquired in form of absorbance or log(1/R) in wavelength from 1000 to 2500 nm (Fig. 1). Spectra data were then enhanced and corrected in order to eliminate noises due to light scattering, background amplifications and temperature changes on the detector. There are several approaches that can be employed as spectra corrections like smoothing, normalization, spectra derivatives and transformations [1]. Two most common used spectra corrections and enhancements are multiplicative scatter correction (MSC) as shown in Fig. 2 and de-trending (DT) as presented in Fig. 3.

**Fig. 4:**
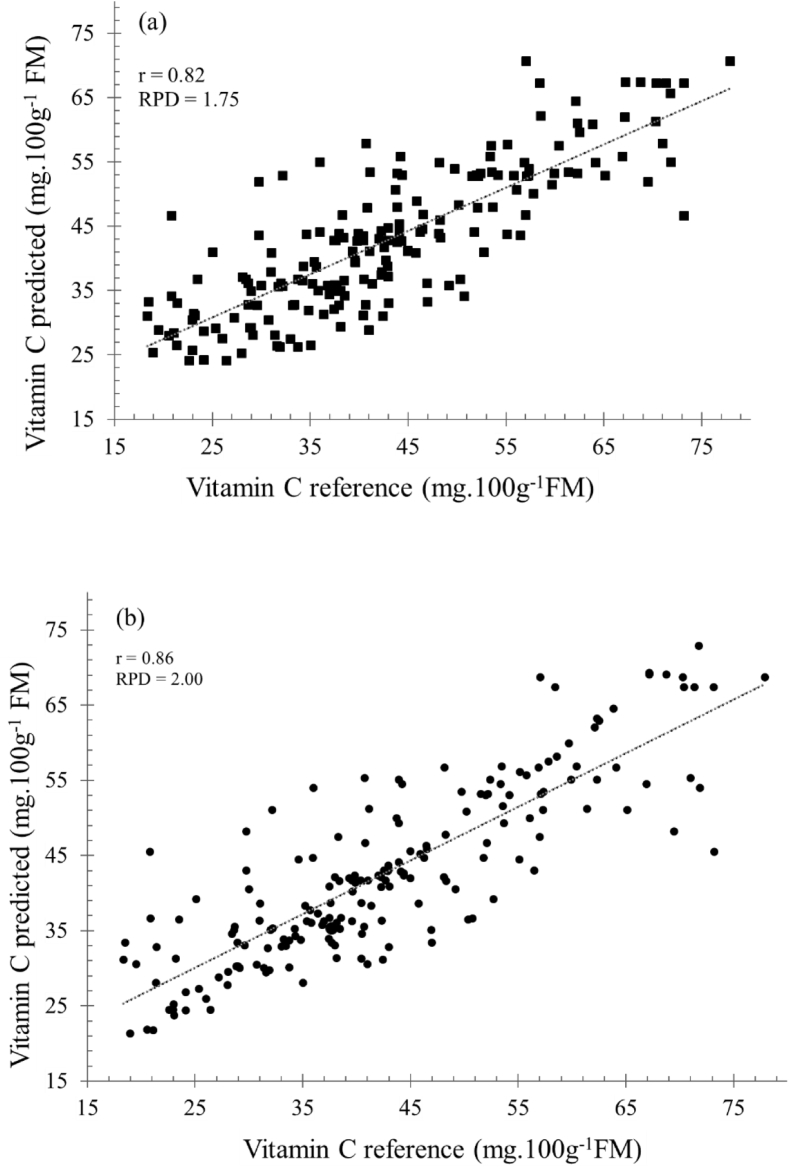
Prediction performance using raw (a) and MSC enhanced (b) dataset to determine vitamin C of intact mango fruits.

Spectra data set were then used to establish prediction model for the determination of inner quality attributes on intact mangos using regression approach. One of the most promising and common regression method in NIRS practices is partial least square regression (PLSR) [2]. This method used to find best correlation between NIR spectra data and respective quality attributes such as vitamin C, SSC and TA of mango fruits. Prediction models were constructed by means of raw and enhanced spectra data as shown in Fig. 4.

## 2 Experimental design, materials, and methods

### 2.1 Instrument preparations

The NIR instrument used to obtain spectral data is Fourier transform near infrared (FT-NIR) (Thermo Nicolet Antaris II TM). It was configured and controlled under integrated software Thermo Integration® and Thermo Operation®. The workflow has been developed and set up to run specified tasks for spectra data acquisition of intact mango samples. High resolution measurement with integrating sphere was chosen as a basic measurement [3].

Sample naming and labelling was required automatically for each spectra acquisition in order to distinct all 186 mango samples. Background spectra correction was carried out every hour automatically. Spectra data were recorded in form of absorbance (Log(1/R)) in wavelength range from 1000 to 2500 nm and saved in two different file extension formats: Nicolet (*.spa*) and comma separated value (*.csv*). Standard laboratory method also prepared for inner quality attributes measurements of mango as vitamin C, soluble solids content (SSC) and total acidity (TA). Hand held refractometer (model HRO32, *Krüss Optronic* GmbH) was used for SSC, automatic titrator equipment (Titroline 96, Schott) for TA and standard manual titration method for vitamin C measurement respectively. The centrifuge was also used to obtain clarified sample juice and separate suspended solids.

### 2.2 Spectra data acquisition

**Fig. 5:**
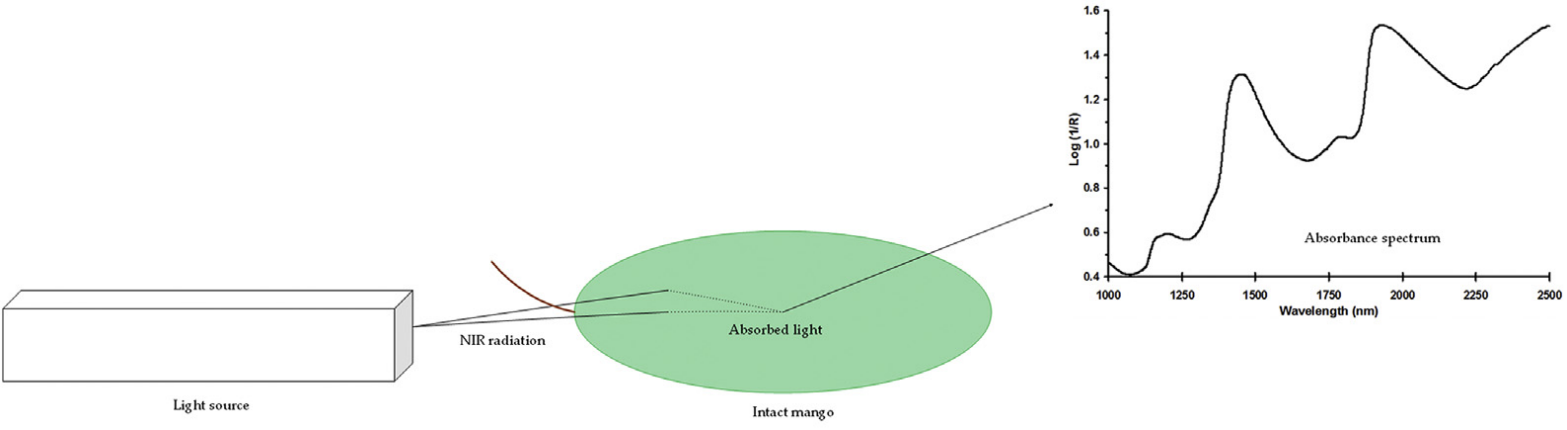
Near infrared spectra data acquisition for intact mango.

Near spectra data were acquired firstly using the FT-NIR instrument. Intact mango sample were placed centrally upon the fruit holder as illustrated in Fig. 5. Each single fruit was hand placed right to the incoming holes (1 cm of diameter) of the light source to ensure direct contact and minimize noises due to light scattering. Absorbance spectra (Log 1/R) in wavelength range of 1000–2500 nm were acquired with co-added of 64 number of scans. Spectra data of each fruit sample were measured in six different points and averaged [2].

### 2.3 Vitamin C, SSC and TA measurements

Once after spectra data acquisitions are completed, fruit samples were directly sliced and some part of mango pulp were taken. To measure vitamin C, a total 5g of mango pulp samples were macerated, mixed and homogenized with addition of 20 ml of meta-phosphoric acid into a beaker to prevent oxidation. Distilled water were added until 50 ml of volume was reached. Vitamin C was obtained and quantified based on its reaction with the *Dichlorophenolindophenol* as an indicator in titration method from which colour change from colourless to light red at the end of titration. Vitamin C of intact mango fruit are expressed in mg∙100g^−1^ fresh mass (FM) [4].

On the other hand, SSC and TA quality attributes were measured by making another juices from 20 g of pulp sample and maximum of 100 ml distilled water. A little filtered juice was squeezed and dropped into a hand-held refractometer to record SSC in form of ^o^Brix while automatic titration method with 0.1 N NaOH to an end point of pH 8.1 was used to measure TA of intact mango and expressed as mg∙100g^−1^ fresh mass [5,6]. Descriptive statistics of actual vitamin C, SSC and TA measurements of mango samples is shown in Table 1.

**Table 1:** Descriptive statistics of actual measured quality attributes of mango samples.

Statistical parameters	Quality attributes		
	Vitamin C	SSC	TA
Number of samples	186	186	186
Mean	42.78	15.76	489.45
Max	77.86	25.51	993.83
Min	18.33	7.91	111.67
Range	59.54	17.60	882.17
Std. Deviation	13.69	3.91	167.42
Variance	187.32	15.25	28028.35
RMS	44.91	16.23	517.15
Skewness	0.41	0.15	−0.01
Kurtosis	−0.44	−0.58	−0.18
Median	40.99	15.42	491.74
Q1	33.05	13.11	369.50
Q3	52.13	18.66	621.23

### 2.4 Spectra data enhancement

It is highly recommended to perform spectra correction and enhancement prior to prediction model development. There are several methods and approaches that can be used to enhance spectra data, among of them are Multiplicative scatter correction (MSC) and de-trending (DT). Spectra enhancements were used to correct additive and multiplicative effects in the spectra. The MSC and DT spectra corrections were carried out using *The Unscrambler X 10.1* software (CAMO, Oslo Norway) with network client license.

### 2.5 Quality attributes (Vitamin C, SSC and TA) prediction

The main part of NIRS application is to develop models used to predict desired quality attributes of intact mango simultaneously. Prediction models were constructed to reveal information about mango quality attributes buried in absorbance spectra data. These models were developed commonly by regressing spectra data (as X variable) and actual measured quality attributes (as Y variable) through multivariate analysis. Partial least squares regression (PLSR) was applied to develop those models and validated using full cross validation approach.

The prediction performances were evaluated by means of these following statistical parameters: the coefficient of correlation (r) and determination (R^2^) between predicted and measured quality attributes, prediction error which is defined as the root mean square error (RMSE) and the residual predictive deviation (RPD), defined as the ratio between standard deviation (SD) of the population's actual value of Vitamin C, SSC and TA, and the RMSE of predicted quality attributes. The higher value of RPD, the greater probability of models to predict desired quality parameters or chemical constituent of samples dataset accurately [[7], [8], [9]].
